# Long‐Term Creatine Supplementation Improves Cognitive and Hippocampal Structural Plasticity Impairments in a D‐Gal‐Induced Aging Model via Increasing CK‐BB Activity in the Brain

**DOI:** 10.1002/fsn3.4767

**Published:** 2025-01-15

**Authors:** Zhu Zhu, Hantao Zhang, Qianlin Li, Xu Han, Tiantian Wang, Wenjing Zhang, Feiyan Chen, Ling Gu, Qi Yao, Lin Chen, Yunan Zhao

**Affiliations:** ^1^ Department of Pathology and Pathophysiology, School of Medicine Nanjing University of Chinese Medicine Nanjing China; ^2^ Department of Physiology, School of Medicine Nanjing University of Chinese Medicine Nanjing China; ^3^ Research and Innovation Center, College of Traditional Chinese Medicine Nanjing University of Chinese Medicine Nanjing China; ^4^ Integrated Chinese and Western Medicine College Nanjing University of Chinese Medicine Nanjing China

**Keywords:** cognitive impairment, creatine kinase BB, creatine supplementation, D‐galactose, structural plasticity

## Abstract

Creatine (Cr) is recognized for its role in enhancing cognitive functions through the phosphocreatine (pCr)–creatine kinase system involved in brain energy homeostasis. It is reversibly converted into pCr by creatine kinase (CK). A brain‐specific isoform of CK, known as CK‐BB, is implicated in the brain's energy metabolism. The objective of this research is to ascertain the impact of Cr supplementation on learning and memory skills as well as on structural synaptic plasticity, by modulating CK‐BB. First, we utilized various concentrations of D‐galactose (D‐gal) to create an aging mouse model. Our findings indicated that D‐gal injections at 100 and 1000 mg/kg could lead to cognitive decline, oxidative stress, and damage to structural synaptic plasticity. CK‐BB expression and its activity were reduced at least by approximately 20% in mice injected with 100 and 1000 mg/kg D‐gal compared with control group. Next, an adeno‐associated virus directed against CKB was employed to reduce CK‐BB levels by 34% in the brain. The reduction of CK‐BB in the brain resulted in deficits in learning and memory, oxidative stress, and morphological harm to the hippocampal spines of mice. Finally, the diet of the D‐gal‐induced aging model was enriched with 3% Cr. Mice that received 3% Cr supplementation exhibited a 36% increase in CK‐BB activity and a 14.3% increase in CK‐BB expression following prolonged D‐gal administration. In addition, Cr supplementation mitigated the cognitive impairment, oxidative stress, and hippocampal structural plasticity damage caused by chronic D‐gal injections. Overall, our study revealed that CK‐BB has a critical role in mediating structural plasticity in D‐gal‐induced cognitive impairment. Moreover, it showed that supplementary Cr could serve as a potent neuroprotective substance, preventing or delaying the course of age‐related cognitive deficits.

## Graphical abstract

Supplementary Creatine could serve as a potent neuroprotective substance, preventing or delaying the course of age‐related cognitive deficits by regulatine CK‐BB.

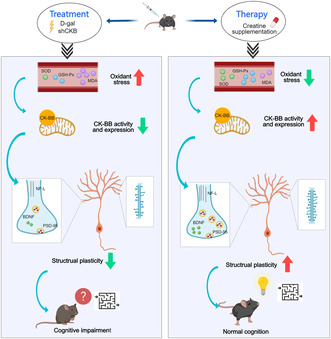

## 1 Introduction

Aging is a complex, time‐dependent process characterized by a gradual decline in the body's physiological functions, which can result in a reduced ability to perform daily activities and a lower overall quality of life (Lopez‐Otin et al. 2023). The United Nations' World Population Prospects predicts that the percentage of individuals who are 60 years of age or over will rise from 12% in 2015 to 22% by 2050 (Azman and Zakaria 2019). A cardinal feature of aging is brain aging, which is particularly susceptible to the effects of aging. This susceptibility can culminate in a decline in cognitive abilities, including a decline in learning and memory, attention span, decision‐making speed, sensory perception, and motor coordination (Mattson and Arumugam 2018). The age‐associated deterioration of cognitive functions is correlated with alterations in the central nervous system, particularly in areas such as the prefrontal cortex and hippocampus, which can contribute to the onset of neurodegenerative conditions like Alzheimer's disease (AD) and dementia. Age‐related cognitive impairments are also associated with impaired motor coordination, leading to a loss of autonomy and a diminished quality of life. The WHO has identified dementia as a significant public health issue, as it is a leading cause of disability and dependency among older adults globally (Wortmann 2012). Therefore, finding effective antiaging therapeutic interventions, allowing researchers to manage the socioeconomic burden of age‐related disorders, is urgently needed.

Creatine (Cr), chemically known as *N*‐[aminoiminomethyl]‐*N*‐methyl glycine, is a naturally occurring compound that is both ingested through food and produced in the body by the liver, pancreas, and kidneys (Walker 1979). It can be transformed into phosphocreatine (pCr) by the action of Cr kinase (CK), which facilitates a reversible reaction between Cr and adenosine triphosphate (ATP) to form pCr and adenosine diphosphate (ADP) (Beard and Braissant 2010). The Cr/PCr system is crucial for sustaining energy levels across various tissues, particularly in those that require high energy expenditure, such as skeletal muscle, heart, and brain. Different isoforms of CK ensure that ATP is efficiently channeled from areas of production to areas of high consumption. CK BB (CK‐BB) is a brain‐specific isoform of CK, is critical for energy metabolism within the brain (Beard and Braissant 2010). Cr, as a substrate of CK, is an ergogenic supplement that is widely used by athletes to enhance their physical performance (Tarnopolsky 2010). In the last decade, a multitude of studies have shown that Cr supplementation can be beneficial in neurological diseases. Randomized clinical trials have indicated that oral Cr supplementation can enhance memory performance, especially in older adults aged 66–76 (Avgerinos et al. 2018). Individuals with genetic Cr disorders characterized by mental dysfunction, including arginine–glycine amidinotransferase (AGAT), guanidinoacetate methyltransferase (GAMT), or solute carrier family 6 member 8 (SLC6A8) deficiencies, have also been shown to benefit from early Cr supplementation (Schulze and Battini 2007). Furthermore, Cr has demonstrated neuroprotective properties, enhancing cognitive function in various animal models. For instance, in female albino mice, Cr supplementation over a 10‐week period was found to protect against learning and memory impairments following neonatal hypoxia‐ischemia (Allah Yar, Akbar, and Iqbal 2015). In a mouse model of AD, Cr supplementation improved spatial cognition, with effects on Aβ and tau protein processing, and increased the expression of several proteins related to neural plasticity (Snow et al. 2020). The evidence suggested that Cr could modulate cognitive functions which indicating its potential for therapeutic or preventative applications. However, the precise mechanisms by which Cr influences cognitive processes remain to be fully elucidated.

Consequently, this study was conducted to investigate whether creatine supplementation could counteract the cognitive deficits, specifically learning and memory impairments, caused by D‐galactose (D‐gal) administration, potentially through the modulation of CK‐BB activity.

## 2 Materials and Methods

### 2.1 Animals

Male C57BL/6J mice, weighing between 18 and 22 g, were procured from the Laboratory Animal Center of Nanjing University of Chinese Medicine. Upon arrival, the mice were housed under consistent environmental settings (a room temperature maintained at 22°C with a variation of ±2°C, a relative humidity of 55% with a fluctuation of ±5%, and a light/dark cycle of 12 h each). They had unrestricted access to water and food, and they were given a week to acclimate to the lab conditions prior to the commencement of the experimental procedures. The study adhered to the experimental animal welfare guidelines set forth by the Laboratory Animal Research Institute and all protocols and procedures were endorsed by the Institutional Animal Care and Use Committee of Nanjing University of Chinese Medicine (permit number: 202209A029).

### 2.2 Experimental Design

#### 2.2.1 Experiment 1

The first experiment was designed to explore the effects of different doses of D‐gal on cognitive behavior, oxidative stress, the structural plasticity of hippocampal neurons, and the activity of CK‐BB. Mice were randomly divided into three groups (*n* = 15/group) as follows: (1) control group, (2) 100 mg/kg group, and (3) 1000 mg/kg group. D‐gal (purified via HPLC to a purity of ≥ 99%, Sigma, St. Louis, MO, the USA) was dissolved in 0.9% saline solution at a concentration of 100 or 1000 mg/kg and subcutaneously injected into mice daily for 8 weeks to induce cognitive impairments (Sadigh‐Eteghad et al. 2017; Zhang et al. 2023). Meanwhile, the mice in the control group were injected with the vehicle. After behavioral tests, mice were sacrificed for antioxidant and CK‐BB activity assays and Western blot and morphological analyses.

#### 2.2.2 Experiment 2

The second experiment was designed to examine the effects of CK brain‐type (CKB) deficiency in the hippocampus on cognitive behavior, oxidative stress, and the structural plasticity of hippocampal neurons. Mice were randomly divided into four groups (*n* = 15/group) as follows: (1) control group, (2) control + D‐gal group, (3) shCKB (knockdown of CKB in neurons) group, and (4) shCKB + D‐gal group. Mice were subjected to stereotactic surgery under 1% pentobarbital sodium anesthesia and placed in a stereotaxic instrument (ALCBIO, Shanghai, China). The skull surface of a mouse was exposed, and the right hippocampus was injected with the control adeno‐associated virus (AAV) containing a negative control (NC) sequence or AAV containing the shRNA sequence targeting CKB. An AAV solution, with a volume of 0.5 μL, was administered into the right hippocampal region at a rate of 0.5 μL/min, using the following stereotaxic coordinates relative to the bregma: AP, −1.7 mm; MR, −1.3 mm; and dorsal ventral (DV), −2 mm. The injection needle was left in position for 10 min post‐injection to prevent backflow. Upon withdrawal of the microsyringe, the skull opening was sealed using dental cement, and the incision in the scalp was sutured closed. After the surgical operations were finished, the mice were placed back into their home cages to allow for optimal gene expression for a minimum of 3 weeks. Thereafter, the mice received subcutaneous injections of 100 mg/kg of D‐galactose for a period of 8 weeks. The accuracy of the injection site was verified by dissecting the brain and observing the intrinsic enhanced green fluorescent protein (EGFP) fluorescence under a fluorescence microscope (Figure 3B). The silencing efficiency of shRNA against CKB was assessed on the basis of our previous study (Zhu et al. 2024). The cognitive behavior of mice was observed, and mice were then sacrificed for antioxidant and CK‐BB activity assays and Western blot and morphological analyses. The detailed experimental procedure is shown in Figure 3A.

#### 2.2.3 Experiment 3

The third experiment was designed to determine the therapeutic effects of Cr supplementation on D‐gal‐induced cognitive impairments and hippocampal neuron damages in the mouse model. Mice were randomly assigned to four groups (*n* = 15/group) as follows: (1) control group, (2) D‐gal group, (3) Cr group, and (4) Cr + D‐gal group. Cr‐supplemented diets were purchased from Jiangsu Xietong Pharmaceutical Bioengineering Co. Ltd. (Nanjing, China) and supplemented into the diets of the Cr and Cr + D‐gal groups at the rate of 3% for eight consecutive weeks. The control and D‐gal groups were fed a normal rodent diet. Mice were injected subcutaneously with 100 mg/kg D‐gal in accordance with their corresponding groups for 8 weeks. The control group received the subcutaneous administration of the vehicle. After the behavioral tests, mice were sacrificed for antioxidant and CK‐BB activity assays and Western blot and morphological analyses.

### 2.3 Behavioral Tests

#### 2.3.1 Y‐Maze

The Y‐maze test was utilized to assess short‐term memory in mice, following 8 weeks of pharmacological intervention, with the aid of a DigBehav animal behavior video analysis system (Shanghai Jiliang Software Technology Co. Ltd., Shanghai, China) (Kraeuter, Guest, and Sarnyai 2019). The maze used for the test was a light‐gray polyvinylchloride Y‐shaped structure consisting of three arms of equal dimensions (each arm being 20 cm in length, 10 cm in width, and 20 cm in height), designated as the start, novel, and familiar arms. The arms intersected at angles of 120°. The test protocol was split into two phases: a training session and a testing session. During the training phase, the mouse was positioned in the starting arm, facing the center of the Y‐maze, with the novel arm blocked, and was permitted to explore the maze freely for a duration of 5 min. After the training trial and before the testing trial, the mouse was returned to its home cage for a rest period of at least 3 h. In the testing phase, the mouse was given the freedom to explore all three arms of the maze for a period of 3 min, with the novel arm now accessible. A video camera recorded the number of entries into each arm and the movement patterns of each mouse. The mice's curiosity towards the new environment was quantified by calculating the ratio of entries into the novel arm to the entries into the familiar arm.

#### 2.3.2 Morris Water Maze (MWM)

The water maze test was utilized in a series of trials to assess the spatial learning and memory capabilities of mice. Each mouse was introduced into one of the four quadrants of a black pool, which had a diameter of 100 cm and was filled with water to a depth of 40 cm, maintained at a temperature of 21°C with a variance of 1°C. The pool was sectioned into four areas: north, south, west, and east. A submerged cylindrical platform, with a diameter of 7 cm and positioned 1–1.5 cm beneath the water's surface, was in the north quadrant. The mice's movements were tracked by a camera mounted above the maze.

During the experiment, each mouse was randomly placed in one quadrant and given 60 s to swim. They underwent four training sessions daily for 5 days, with a 30‐min break between each session. The time taken (in seconds) for each mouse to locate the platform was documented. If a mouse failed to find the platform within the allotted time, the researcher would guide it to the platform, where it would stay for 15 s. On the sixth day, a probe trial was conducted without the platform. The mouse was released from the quadrant opposite to where the platform had been, and it was allowed to swim for 60 s. The percentage of swimming time spent in the target quadrant, number of mice passing over the quondam platform, and swimming speed were analyzed by using a DigBehav animal behavior video analysis system (Shanghai Jiliang Software Technology Co. Ltd., Shanghai, China).

### 2.4 Data Normalization and Identification of Differentially Expressed Genes

The Gene Expression Omnibus (GEO) database was used to obtain microarray data from the brain specimens of patients suffering from AD, cognitive impairments, or dementia. The GPL18573 datasets of GSE193438, which included frozen slices from four brain areas (substantia nigra, hippocampus, parietal lobe, and basal ganglia) of patients with AD and nondemented controls, were screened in this experiment. The original files downloaded from the GEO database were preprocessed and normalized. A volcano plot was used to enhance the visualization of differentially expressed genes (DEGs). The screening criterion was *p*‐value (−log_10_) < 0.05.

### 2.5 Construction of the Viral Vector for CKB Knockdown

To achieve knockdown labeling of CKB in neurons, the viruses rAAV‐hsyn‐EGFP‐5′miR‐30a‐shRNA (Ckb)‐3′‐miR30a‐wpres (AAV2/9, ≥ 2 × 10^12^ vg/mL) and its control viruses rAAV‐hsyn‐EGFP‐5′miR‐30a‐shRNA (scramble)‐3′‐miR30a‐wpres (AAV2/9, ≥ 2 × 10^12^ vg/mL) were produced by Wuhan BrainVTA Co. Ltd., China. Briefly, three shRNA sequences were designed to target CKB (gene ID 24264), and the hairpin plasmid that exhibited the most significant knockdown in vitro was selected for packaging and utilized in all subsequent experiments (5′‐GGGTTATCTCCATGCAGAAAG). The non‐targeting control (NC) sequence was 5′‐CCTAAGGTTAAGTCGCCCTCG. The presence of AAV infection in the hippocampi of mice was determined by the expression of EGFP. Following the viral injection as described in experiment 2, mice were euthanized and perfused with 4% paraformaldehyde. The brain tissues were then extracted and sectioned serially at 25 μm thickness using a freezing microtome (Histo‐Line Laboratories, Italy). The sections at the site of injection were treated with antifade mounting medium containing DAPI (Beyotime, Shanghai, China) and examined for green fluorescence using a Zeiss Axio Imager M2 fluorescence microscope (Figure 3B).

### 2.6 Antioxidant Activity Assay

Following the behavioral assessments, blood samples were obtained from mice by enucleation under anesthesia induced by 1% sodium pentobarbital. The serum was promptly collected and preserved at a temperature of −70°C. Diagnostic kits for the measurement of superoxide dismutase (SOD), glutathione peroxidase (GSH‐Px), and malondialdehyde (MDA) were sourced from Jiancheng Institute of Biotechnology (Nanjing, China). The experimental procedures were carried out strictly following the protocols provided by the kit manufacturers.

### 2.7 CK‐BB Activity Assay

Mice were euthanized by decapitation under deep anesthesia induced by pentobarbital sodium, and their hippocampi were swiftly dissected on ice. Brain sections were homogenized in nine volumes of 0.9% saline by using an ultrasonic machine. The resulting homogenate was subjected to centrifugation at 2500 rpm for a duration of 10 min at a temperature of 4°C. The supernatant was subsequently diluted to a concentration of 2% for the activity assay. The activity of CK‐BB within the hippocampus was measured using a colorimetric method with a microplate reader (Allsheng, Hangzhou, China) and a CK assay kit (Jiancheng Institute of Biotechnology, Nanjing, China), following the instructions provided by the manufacturer.

### 2.8 Western Blot Analysis

The hippocampus was processed by homogenization in lysis buffer and then centrifuged at 12,000 rpm for 15 min at a temperature of 4°C. The concentration of protein was measured using a BCA protein assay kit (Cwbio, Jiangsu, China) following the protocol provided by the manufacturer. Protein samples of equivalent amounts were resolved by 10% SDS‐polyacrylamide gel electrophoresis and transferred onto PVDF membranes. The membranes were then blocked with a solution of 5% nonfat milk for a period of 2 h at room temperature. The primary antibodies used included: mouse monoclonal anti‐β‐actin (1:5500, Proteintech), rabbit monoclonal antibrain‐derived neurotrophic factor (BDNF, 1:1000, Abcam), rabbit monoclonal anti‐CKB (1:1000, Abcam), mouse monoclonal antineurofilament light polypeptide (NF‐L, 1:1000, Invitrogen), and rabbit monoclonal anti‐PSD‐95 (PSD‐95 1:3000, Proteintech). After three washes with TBST, the membranes were incubated with the appropriate secondary antibodies, either goat anti‐mouse IgG (1:5000, Proteintech) or goat antirabbit IgG (1:5000, Proteintech) for 1 h at room temperature. The protein bands were made visible using an ECL Western blot detection system. Chemiluminescent signals were recorded with image analysis software (NIH), and the relative protein levels were expressed as the ratio of the protein of interest to β‐actin.

### 2.9 Golgi–Cox Staining

Golgi–Cox staining was employed to observe dendritic branching patterns and spines. The brains collected were processed using the Hito Golgi–Cox OptimStain PreKit (Hitobiotec Corp., Kingsport, TN, the USA), with all staining procedures strictly following the manufacturer's guidelines. In summary, the brain was immersed in the Golgi–Cox solution for a period of 14 days in darkness at ambient temperature. After this, the brain was sectioned into 180 μm thick slices using a freezing microtome (Histo‐Line Laboratories, Italy), targeting the hippocampal region, and the sections were then mounted onto gelatin‐coated slides. Ultimately, the brain sections underwent dehydration before being stained and sealed with resin‐sealing tablets. Hippocampal neurons in the CA1 region (*N* = 3–5 mice per group, with *n* = 3 neurons selected at random per mouse) were visualized using a Zeiss Axio Imager M2 microscope. Quantitative assessments of dendritic spine density and total dendritic length measurements and Sholl analysis were performed using Neurolucida and Stereo Investigator (MBF Bioscience, the USA).

### 2.10 Statistical Analysis

The data were displayed as the mean ± SEM. Statistical evaluations were conducted using either one‐way ANOVA or two‐way ANOVA, complemented by a Tukey's multiple comparisons test for post hoc analysis. The data analysis was carried out using GraphPad Prism version 9 (GraphPad Software, the USA). The results were considered statistically significant at a *p*‐value < 0.05.

## 3 Results

### 3.1 Experiment 1

Effects of different doses of D‐gal on cognitive behavior, oxidative stress, CK‐BB activity, and the structural plasticity of hippocampal neurons.

#### 3.1.1 CKB Was Downregulated in the Brain Tissue of Patients With AD

**FIGURE 1:**
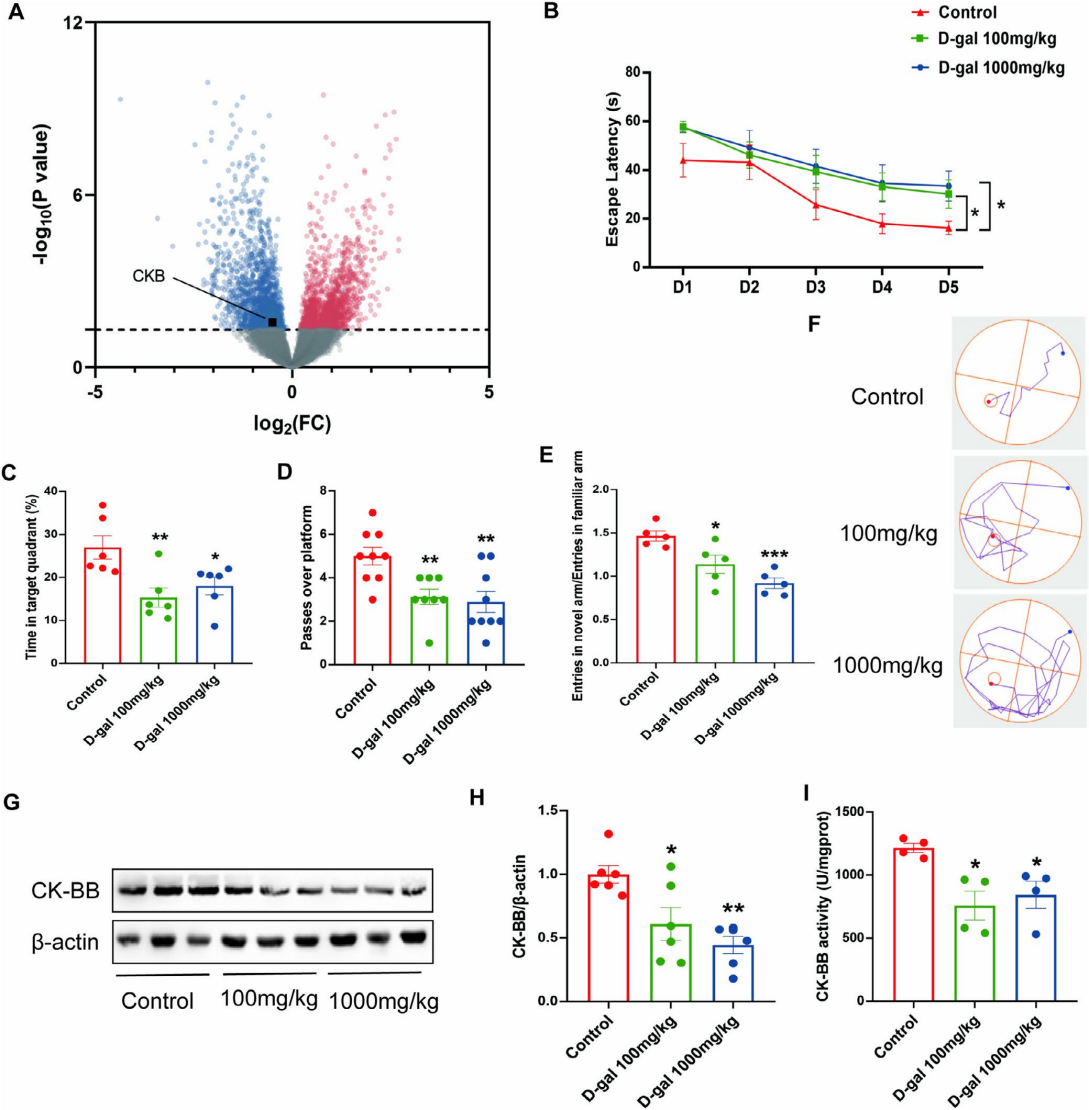
The effects of different doses of D‐gal on cognitive behavior, oxidative stress, and CK‐BB activity. (A) Volcano plot of differentially expressed genes (DEGs) in Alzheimer's disease (AD). Volcano plots were generated based on the fold‐change of gene expression using the averaged spectral counts. The *x*‐axis indicates a log_2_ fold‐change, and the *y*‐axis indicates −log_10_ *p*‐value based on Student's *t*‐test. The horizontal line indicates a *p*‐value < 0.05, the *CKB* was labeled in black square. (B–F) Morris water maze. (B) Escape latency of mice during the five training days. (C) The percentage of time spent in target quadrant in the spatial probe test. (D) Number of crossings to the platform area during the spatial probe trial. (F) Representative images of the trajectory diagram of mice during the training period on the 5th day. (E) Entry number ratio of the new arm to the familiar arm in Y‐maze. (G) Representative Western blot images of CK‐BB and β‐Actin expression levels in the hippocampus. Western blot analysis showing relative protein levels of CK‐BB (H). (I) CK‐BB activity in the hippocampus. *p < 0.05, **p < 0.01, ***p < 0.001 vs. control group; mean ± SEM.

Microarray datasets for brain tissue in cognitive impairments, AD, and dementia from the GEO database were screened to identify the role of CK‐BB in cognitive dysfunction disorders. The GSE193438 dataset, which included different brain areas of patients with AD and nondemented controls, was selected to analyze and identify DEGs. Comparison with genes in the control samples revealed 3288 DEGs in the AD samples. These DEGs comprised 2507 downregulated genes (blue) and 781 upregulated genes (red). Figure 1A shows the DEGs with −log_10_ (*p*‐value) < 10. CKB is one of the downregulated genes marked with black squares.

#### 3.1.2 Injection With Different Doses of D‐Gal Induced Learning and Memory Deficits and Oxidative Stress in Mice

In the MWM test, Figure 1B illustrates that on the fifth day of training, the groups that received 100 and 1000 mg/kg of D‐gal took longer time to locate the hidden platform compared to the control group (*p* < 0.05). Figure 1F presents the representative tracing on day 5 for each group. The control group mice were able to directly locate the hidden platform on day 5. In contrast, the D‐gal‐injected mice followed a series of looping paths to find the platform while swimming in the pool. This observation indicates that the control group had learned the platform's location through training, while the D‐gal‐injected mice seemed to find the platform by chance. Following this, a probe test was conducted by removing the platform to evaluate the strength of the memory trace. The time spent swimming in the target quadrant and the number of platform crossings by the mice in the groups treated with 100 (*p* < 0.01) and 1000 mg/kg (*p* < 0.05, *p* < 0.01) D‐gal were significantly reduced compared to the control group (Figure 1C,D).

In the Y‐maze test, the ratio of entries into the novel arm to entries into the familiar arm in the groups treated with 100 (*p* < 0.05) and 1000 mg/kg (*p* < 0.001) D‐gal significantly reduced compared with those in the control group (Figure 1E).

It has been thoroughly established that prolonged exposure to D‐gal in rodents leads to oxidative stress in the brain (Gong et al. 2008; Zhang et al. 2008). We measured the level of MDA, an indicator of lipid peroxidation, as well as the activities of antioxidant parameters, such as SOD and GSH‐Px, in the hippocampi of mice. Compared to the control group, the groups administered 100 (*p* < 0.01, *p* < 0.001) and 1000 mg/kg (*p* < 0.05, *p* < 0.01) D‐gal showed a reduction in the activities of SOD and GSH‐Px and an elevation in the serum levels of MDA (Figure S1).

The above results indicate that in mice, injections of 100 and 1000 mg/kg D‐gal could successfully induce cognitive deficiency and oxidative stress.

#### 3.1.3 Long‐Term Injection of D‐Gal Reduced the Activity and Protein Level of CK‐BB in the Hippocampi of Mice

Mouse hippocampal tissues were utilized to evaluate the activity and expression levels of CK‐BB following the completion of behavioral tests. The results indicated that administration of 100 (*p* < 0.05) and 1000 mg/kg (*p* < 0.05, *p* < 0.01) D‐gal significantly reduced both the protein expression and activity of CK‐BB (Figure 1G–I).

#### 3.1.4 Long‐Term Injection of D‐Gal Reduced the Hippocampal Structural Plasticity of Neurons in Mice

**FIGURE 2:**
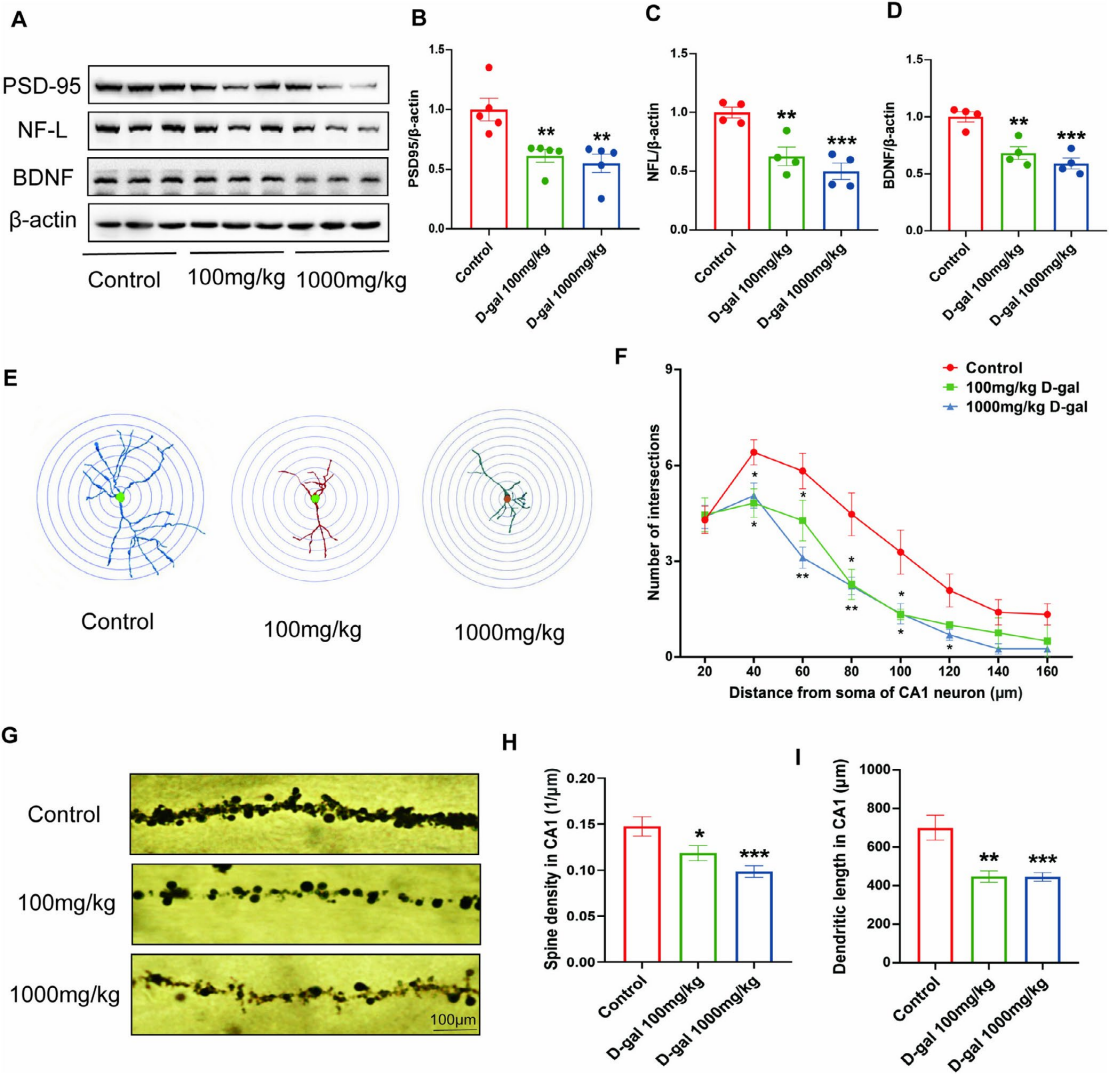
The effects of different doses of D‐gal on structural plasticity of hippocampal neurons. (A) Representative Western blot images of PSD‐95, NF‐L, BDNF, and β‐Actin expression levels. Western blot analysis showing relative protein levels of PSD‐95 (B), NF‐L (C), and BDNF (D). (E, F) Sholl analysis in the CA1 region of hippocampus. (E) Representative images of dendritic tracings of neurons. (F) Number of dendritic intersections. (G) Representative images of dendritic spines, (H) spines density and (I) dendritic length in the CA1. *p < 0.05, **p < 0.01, and ***p < 0.001 vs. control group; mean ± SEM.

To further investigate whether cognitive decline was linked to a reduction in synaptic proteins, the expression levels PSD‐95, NF‐L, and BDNF were assessed using Western blot analysis. The findings demonstrated that 100 (*p* < 0.01) and 1000 mg/kg (*p* < 0.01, *p* < 0.001) D‐gal treatment significantly reduced the protein levels of PSD95, NF‐L, and BDNF in comparison with the control treatment (Figure 2A–D).

Synaptic plasticity can be assessed through both quantitative and qualitative assessments of neuronal structure, particularly focusing on dendritic branching and the characteristics of dendritic spines. Sholl analysis, as well as measurements of spine density and total dendritic length, was utilized to examine the morphological alterations in Golgi‐stained CA1 hippocampal pyramidal neurons. This approach was employed to further investigate the impact of varying doses of D‐gal on hippocampal structural plasticity. Sholl analysis, which represents the distribution of dendritic intersections with increasing distance from the cell soma, revealed that treatment with 100 (*p* < 0.05) and 1000 mg/kg (*p* < 0.05, *p* < 0.01) D‐gal significantly decreased the numbers of CA1 dendritic intersections in the circle diameter of 40–120 μm compared with the control treatment (Figure 2E,F). The spine density and total dendritic length of hippocampal neurons in CA1 decreased significantly after injections with 100 (*p* < 0.05, *p* < 0.01) and 1000 mg/kg (*p* < 0.001) D‐gal injections in contrast to those after the control treatment (Figure 2G–I).

These results suggest that in mice, injections of 100 and 1000 mg/kg D‐gal could successfully induce cognitive dysfunction and oxidative stress and damage hippocampal structural plasticity. These results lacked dose dependency. In the following experiments, we injected 100 mg/kg D‐gal into mice to establish a rodent model of cognitive impairment.

### 3.2 Experiment 2

Effects of CK‐BB knockdown in the hippocampus on learning and memory, oxidative stress, and the structural plasticity of hippocampal neurons.

#### 3.2.1 Silencing Efficiency of the shRNAs Against CKB in the Hippocampi of Mice

**FIGURE 3:**
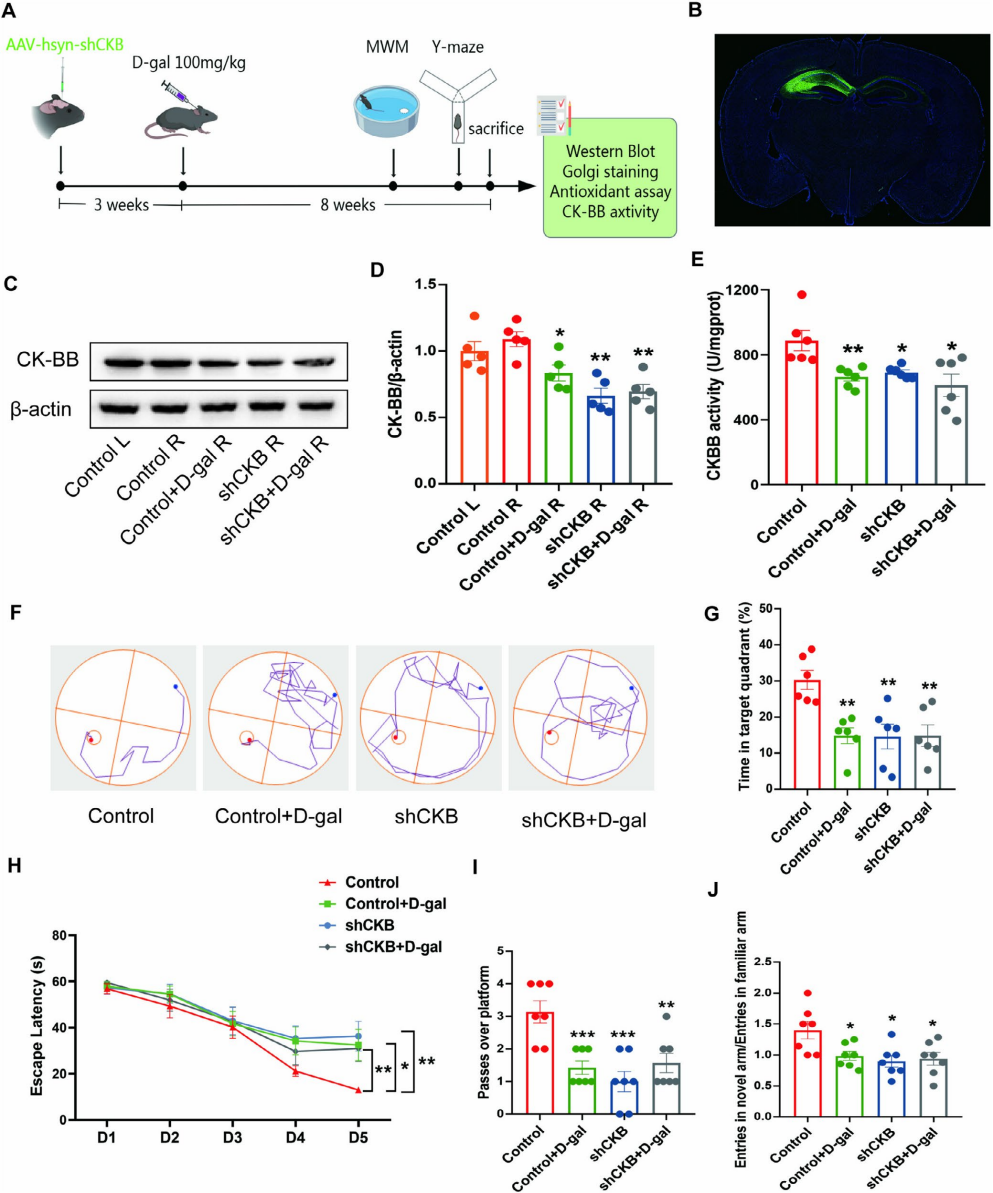
Effects of CK‐BB knockdown in the hippocampus on learning and memory, oxidant stress. (A) Schematic of the experimental paradigm. (B) The right hippocampus of mice injected with control AAV containing a NC sequence. The AAV infection in the hippocampus was observed with eGFP positive expression (green) and DAPI (blue) by IF. (C) Representative Western blot images of CK‐BB and β‐Actin expression levels. Western blot analysis showing relative protein levels of CK‐BB (D). (E) CK‐BB activity in the hippocampus. (F–I) Morris water maze. (F) Representative images of the trajectory diagram of mice during the training period on the 5th day. (G) The percentage of time spent in target quadrant in the spatial probe test. (H) Escape latency of mice during the five training days. (I) Number of crossings to the platform area during the spatial probe trial. (J) Entry number ratio of the new arm to the familiar arm in Y‐maze. *p < 0.05, **p < 0.01 and ***p < 0.001 vs. control group; mean ± SEM.

We injected AAV‐shCKB into the hippocampus by using a stereotaxic technique to knock down CK‐BB expression in the mouse hippocampus and thus detect whether CK‐BB in neurons plays a role in learning and memory skills and hippocampal structural plasticity. The precise positioning of the vectors in mice was verified by removing the brains and observing the EGFP fluorescence under a fluorescence microscope (Figure 3B). As shown in Figure 3C–E, the knockdown of CK‐BB significantly decreased the protein level and activity of CK‐BB compared with the control (*p* < 0.05, *p* < 0.01). All the results of two‐way ANOVA analysis were described in Appendix S1.

#### 3.2.2 Knockdown of CK‐BB in the Hippocampus Induced Cognitive Dysfunction and Oxidative Stress in Mice

As shown in Figure 3H, in the MWM test, the escape latency in the control + D‐gal, shCKB, and shCKB + D‐gal groups decreased significantly on day 5 relative to that in the control group (*p* < 0.05, *p* < 0.01). The representative tracing on day 5 revealed that the control + D‐gal, shCKB, and shCKB + D‐gal groups demonstrated a chaotic search pattern when finding the hidden platform, whereas the control group could almost directly find the hidden platform (Figure 3F). Figure 3G–I showed that the control + D‐gal, shCKB, and shCKB + D‐gal groups had a lower time spent in the target quadrant and lower number of passages over the platform than the control group (*p* < 0.01, *p* < 0.001). The Y‐maze test analysis revealed that the control + D‐gal, shCKB, and shCKB + D‐gal groups had less entries into the new arm than the control group (*p* < 0.05) (Figure 3J).

The oxidative stress indicators showed that the control + D‐gal, shCKB, and shCKB + D‐gal groups had significant reductions in SOD and GSH‐Px activities and an increase in MDA level compared with the control group (*p* < 0.05, *p* < 0.01, *p* < 0.001) (Figure S2).

#### 3.2.3 Knockdown of CK‐BB Impaired the Hippocampal Structural Plasticity of Neurons in Mice

**FIGURE 4:**
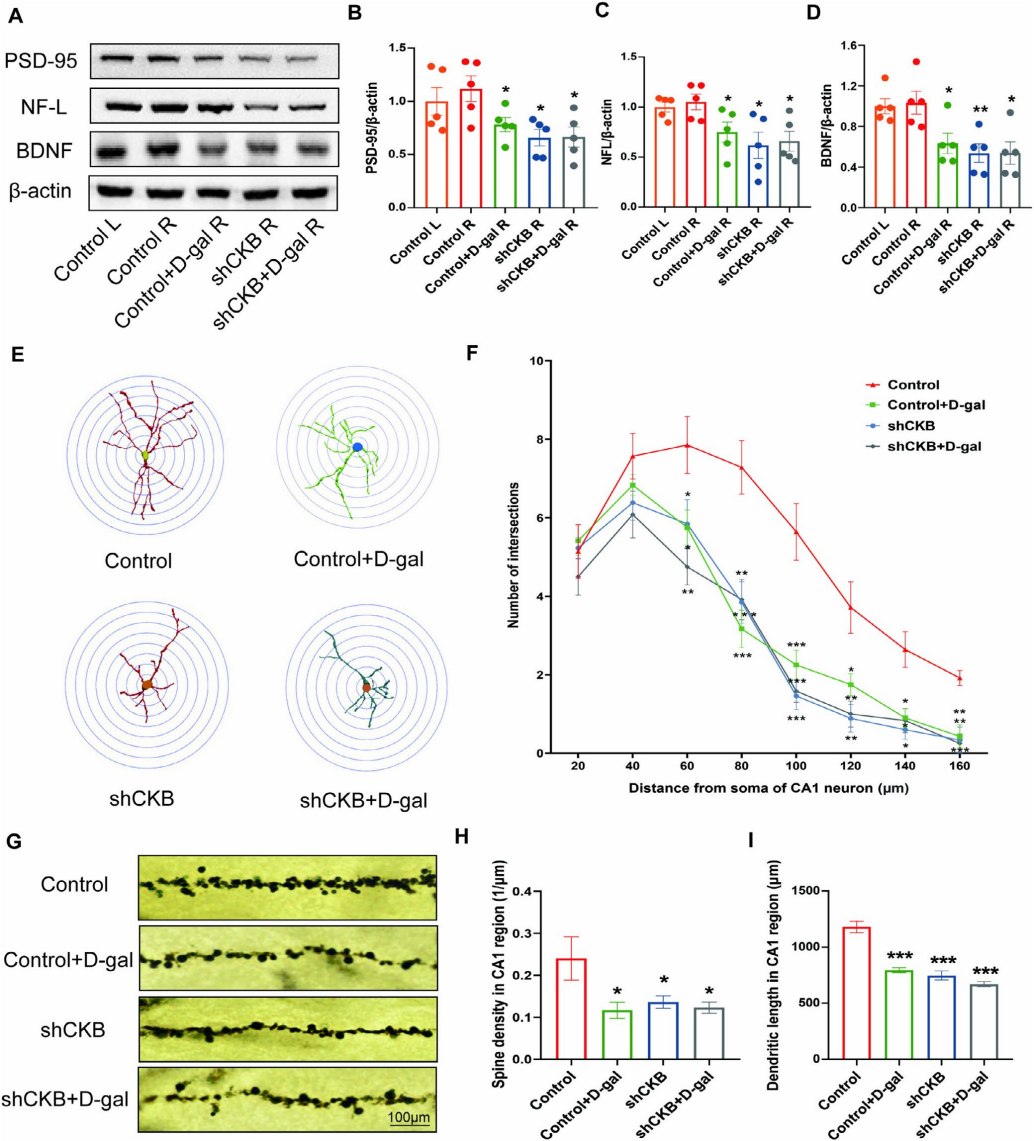
Effects of CK‐BB knockdown in the hippocampus on structural plasticity of hippocampal neurons. (A) Representative Western blot images of PSD‐95, NF‐L, BDNF, and β‐Actin expression levels. Western blot analysis showing relative protein levels of PSD‐95 (B), NF‐L (C), and BDNF (D). (E, F) Sholl analysis in the CA1 region of hippocampus. (E) Representative images of dendritic tracings of neurons. (F) Number of dendritic intersections. (G) Representative images of dendritic spines, (H) spines density and (I) dendritic length in the CA1. *p < 0.05, **p < 0.01, and ***p < 0.001 vs. control group; mean ± SEM.

The western blot results revealed that the control + D‐gal, shCKB, and shCKB + D‐gal groups had significantly decreased protein levels of PSD‐95, NF‐L, and BDNF compared with the control group (*p* < 0.05, *p* < 0.01) (Figure 4A–D).

Sholl analysis showed that the numbers of CA1 dendritic intersections in the circle diameter of 60–160 μm in the control + D‐gal, shCKB, and shCKB + D‐gal groups had significantly decreased compared with that in the control group (Figure 4E,F). The spine density and total dendritic length of hippocampal neurons in CA1 in the control + D‐gal, shCKB, and shCKB + D‐gal groups significantly decreased compared to those in the control group (*p* < 0.05, *p* < 0.001) (Figure 4G–I).

Taken together, these results indicate that the knockdown of CK‐BB in the hippocampus could induce cognitive impairments, oxidative stress, and hippocampal structural plasticity injury.

### 3.3 Experiment 3

Cr supplementation improved cognitive deficits, oxidative stress, and the structural plasticity of hippocampal neurons in the D‐gal‐induced aging mouse model by increasing the activity and protein levels of CK‐BB.

#### 3.3.1 Supplementary Cr in the Diet Relieved Learning and Memory Deficits and Oxidative Stress in Mice

**FIGURE 5:**
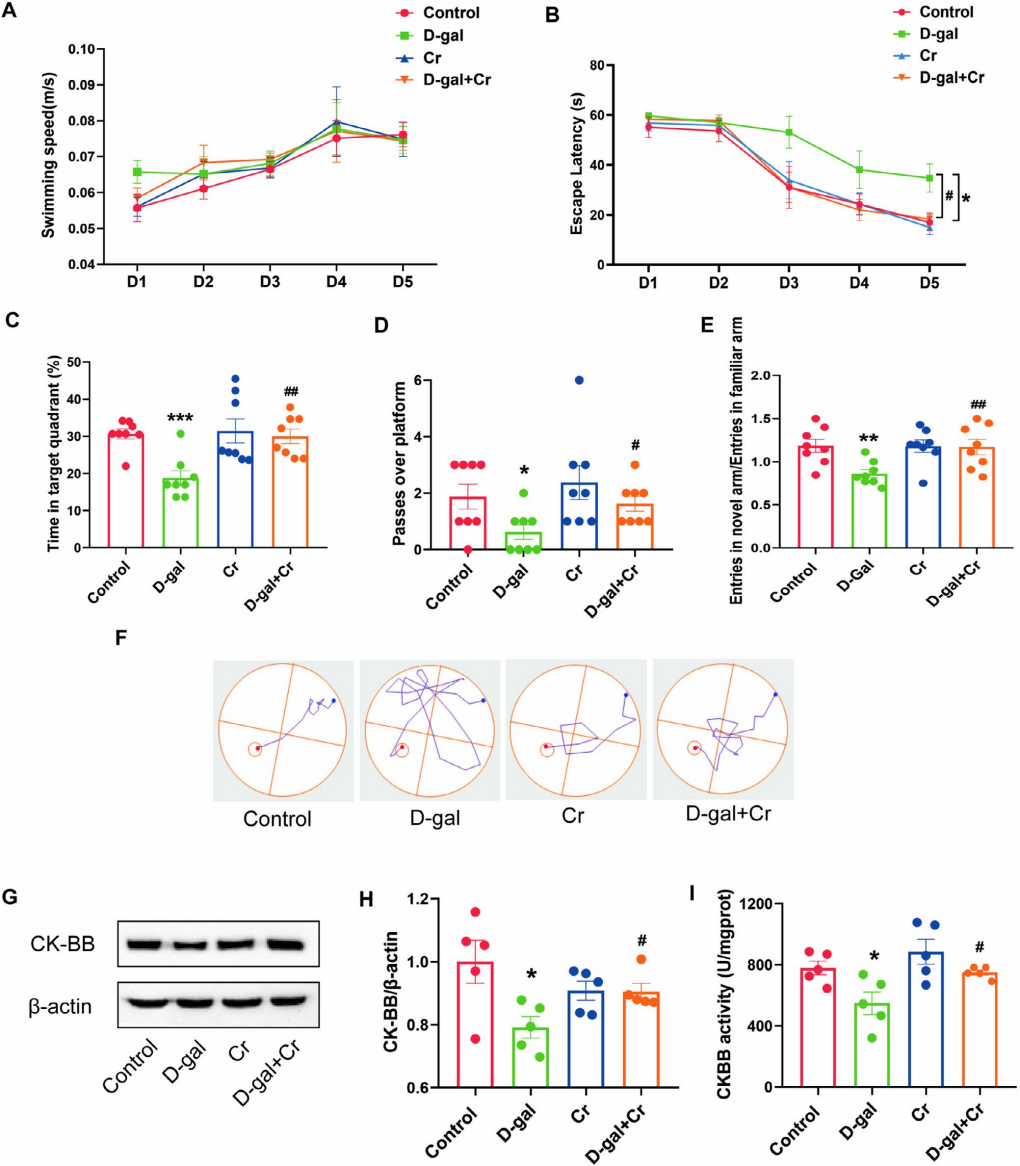
Effects of Cr supplementation on learning and memory, oxidant stress and CK‐BB activity and expression. (A) Swimming speed during five training days in mice (B) Escape latency of mice during the five training days. (C) The percentage of time spent in target quadrant in the spatial probe test. (D) Number of crossings to the platform area during the spatial probe trial. (E) Entry number ratio of the new arm to the familiar arm in Y‐maze. (F) Representative images of the trajectory diagram of mice during the training period on the 5th day. (G) Representative Western blot images of CK‐BB and β‐Actin expression levels. Western blot analysis showing relative protein levels of CK‐BB (H). (I) CK‐BB activity in the hippocampus. *p < 0.05, **p < 0.01, and ***p < 0.001 vs. control group; #*p* < 0.05, ##*p* < 0.01 vs. D‐gal group; mean ± SEM.

As shown in Figure 5A, no difference was found among the swim speeds of all groups during the consecutive 5‐day training test. These results suggest that neither D‐gal treatment nor Cr supplementation for 8 weeks could cause apparent motor and/or visual deficits in mice. The escape latency of the D‐gal group significantly decreased on day 5 compared with that of the control group (*p* < 0.05) (Figure 5B). In contrast to D‐gal treatment, dietary supplementation with 3% Cr significantly lengthened the escape latency in mice on day 5 (*p* < 0.05) (Figure 5B). The representative tracing on day 5 revealed that the control, Cr, and D‐gal + Cr groups could easily find the hidden platform, whereas the D‐gal groups showed looping search patterns while finding the hidden platform (Figure 5F). As shown in Figure 5C,D, long‐term injection of D‐gal resulted in a lower time spent in the target quadrant and lower number of passages over the platform compared with the control treatment (*p* < 0.05, *p* < 0.001). Mice synchronously fed with the Cr diet increased the time spent in the target quadrant and their number of passages over the platform in contrast to the D‐gal‐treated group (*p* < 0.05, *p* < 0.01) (Figure 5C,D). The Y‐maze test analysis revealed that mice injected with D‐gal had less entries into the new arm than the control group (*p* < 0.01), whereas the D‐gal + Cr group had a higher ratio of entries in the new arm to entries into the familiar arm than the D‐gal‐treated group (Figure 5E).

The oxidative stress tests showed that mice injected with D‐gal had a significant decrease in SOD and GSH‐Px activity and an increase in MDA level in contrast to the control group (*p* < 0.05, *p* < 0.01) (Figure S3). In contrast to the D‐gal‐treated group, the D‐gal + Cr group showed a significant increase in SOD and GSH‐Px activity and decrease in MDA level (Figure S3).

#### 3.3.2 Cr Supplementation Increased the Activity and Protein Level of CK‐BB in the Hippocampi of Mice

D‐gal treatment significantly decreased the protein level and activity of CK‐BB (*p* < 0.05). However, these reductions could be antagonized by Cr supplementation (*p* < 0.05) (Figure 5G–I).

#### 3.3.3 Cr Supplementation Improved Hippocampal Structural Plasticity

**FIGURE 6:**
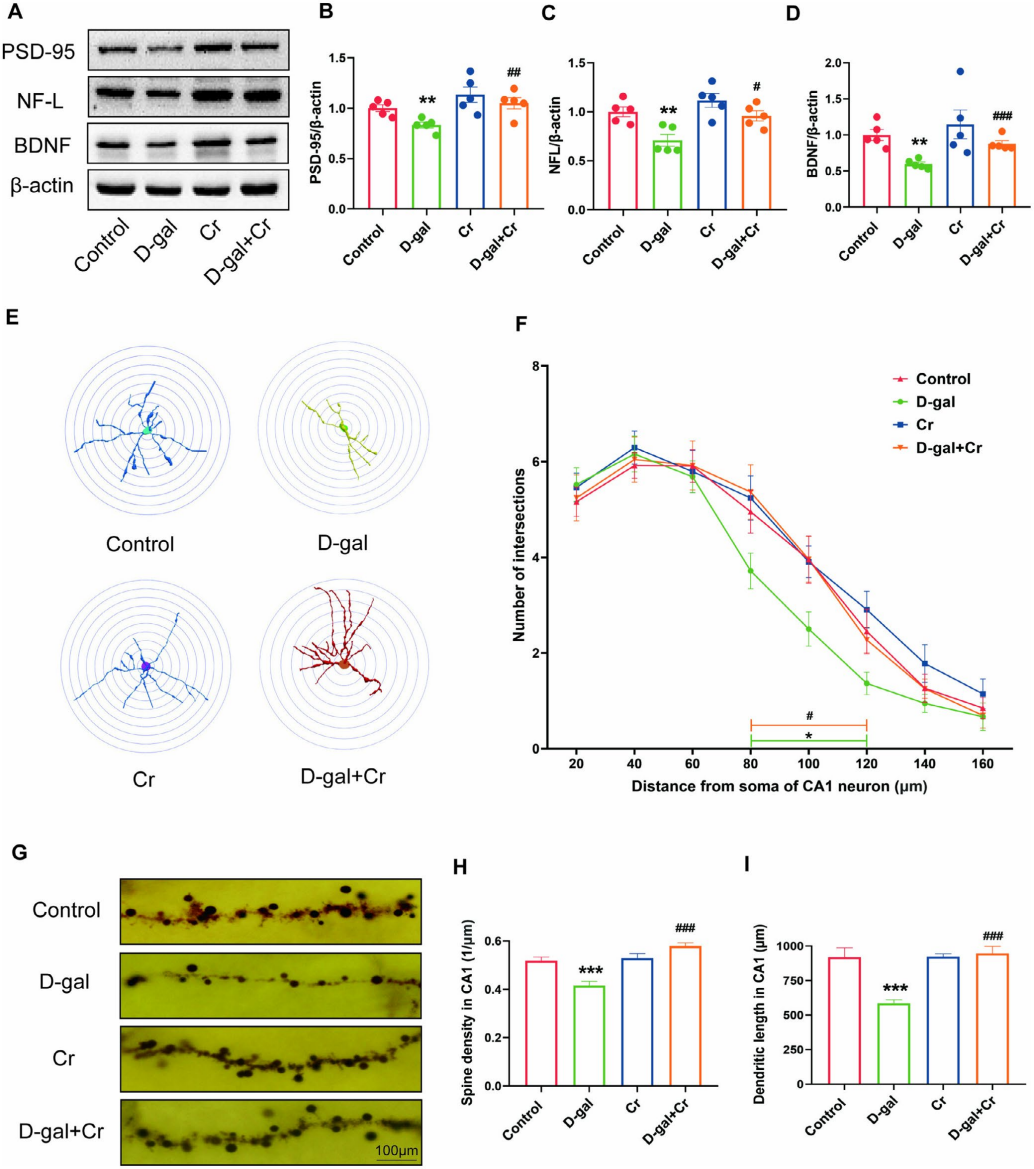
Effects of Cr supplementation in the hippocampus on structural plasticity of hippocampal neurons. (A) Representative Western blot images of PSD‐95, NF‐L, BDNF, and β‐Actin expression levels. Western blot analysis showing relative protein levels of PSD‐95 (B), NF‐L (C) and BDNF (D). (E, F) Sholl analysis in the CA1 region of hippocampus. (E) Representative images of dendritic tracings of neurons. (F) Number of dendritic intersections. (G) Representative images of dendritic spines, (H) spines density and (I) dendritic length in the CA1. *p < 0.05, **p < 0.01, and ***p < 0.001 vs. control group; #*p* < 0.05, ##*p* < 0.01, and ###*p* < 0.001 vs. D‐gal group; mean ± SEM.

Western blot results revealed that the protein levels of PSD‐95, NF‐L, and BDNF decreased significantly in the groups that received long‐term D‐gal injection in contrast to those in the control group (*p* < 0.01). In contrast to D‐gal treatment, supplementation with Cr significantly enhanced the protein levels of synaptic‐related proteins (Figure 6A–D).

Sholl analysis showed that D‐gal significantly decreased the numbers of CA1 dendritic intersections in the circle diameter of 80–120 μm compared with the control treatment (*p* < 0.05), whereas D‐gal + Cr treatment significantly relieved the reduction under D‐gal treatment (*p* < 0.05) (Figure 6E,F). The long‐term injection of D‐gal significantly decreased the spine density and total dendritic length of hippocampal neurons in CA1 (*p* < 0.001) compared to the control treatment. Furthermore, compared with D‐gal treatment, simultaneous feeding with Cr significantly reversed the spine density and total dendritic length of hippocampal neurons in CA1 (*p* < 0.001) (Figure 6G–I).

These results suggest that in mice, the dietary supplementation of Cr could reverse the hippocampal structural plasticity and cognitive impairments that occurred after long‐term D‐gal administration.

## 4 Discussion

### 4.1 Long‐Term D‐Gal Administration Induced Learning and Memory Deficits and Decreased CK‐BB Activity and Expression

Aging is the primary risk factor for diseases associated with age, including those that affect the nervous system. Various animal models have been employed to study the mechanisms behind brain aging. D‐gal, a naturally occurring sugar in the body and brain, is used to simulate the aging process in animals like rodents when administered chronically (Nagy and Pohl 2015). These animals exhibit remarkable reductions in lifespan, cognitive function, and immune response and abnormal biochemistry indicators (Ho, Liu, and Wu 2003; Shang et al. 2001; Shen et al. 2002; Xu and Zhao 2002). After injection, D‐gal can be transformed into aldose and hydroperoxide by the action of galactose oxidase, leading to the production of reactive oxygen species (ROS) (Wu et al. 2008). Elevated ROS levels can lead to oxidative stress, inflammation, mitochondrial dysfunction, and cell death (Ullah et al. 2015). While previous research has shown that D‐gal induces signs of aging, the dosages used across different labs have not been consistent. In our study, we classified D‐gal doses into low (100 mg/kg) and high (1000 mg/kg) based on a systematic review and meta‐analysis of the D‐gal‐induced aging model (Sadigh‐Eteghad et al. 2017). We observed that mice treated with 100 and 1000 mg/kg D‐gal displayed cognitive impairments, along with decreased activities of SOD and GSH‐Px and increased levels of MDA (Figure S1). A reduction in hippocampal synapse count and changes in dendritic structure and dendritic spine morphology can lead to impaired synaptic plasticity. Furthermore, a decrease in the expression of synaptic proteins such as synaptophysin and BDNF may contribute to reduced hippocampal synaptic plasticity with age, exacerbating cognitive decline (Bettio, Rajendran, and Gil‐Mohapel 2017). In the present study, we found that D‐gal administration led to a substantial decrease in spine density and dendritic length in the CA1 region and a reduction in the number of CA1 dendritic intersections within a circle diameter of 40–120 μm (Figure 2E–I). In mice, the expression of synapse‐related proteins, including BDNF, SYP, and NF‐L, could be significantly reduced by injections of 100 and 1000 mg/kg D‐gal (Figure 2A–D). Our findings suggested that prolonged exposure to 100 and 1000 mg/kg D‐gal in mice could mimic an aged state, characterized by increased oxidative stress and subsequent damage to hippocampal synaptic plasticity, resulting in cognitive impairment. There is no significant difference in cognitive ability and oxidative stress indicators between mice injected with 100 and 1000 mg/kg D‐gal in our study. Why does the high dose of D‐gal not aggravate oxidative stress or cognitive dysfunction, possibly because of increased antioxidant defense mechanisms in animals, or increased protection against oxidative damage, or adaptation to increased oxidant proliferation (Serrano and Klann 2004). In accordance with the principle of 3Rs, which aim to decrease the incidence and/or severity of inhumane procedures, minimize pain and distress, and enhance welfare in animals in experimental protocols (Diaz et al. 2020), we used mice injected with 100 mg/kg D‐gal as an aging animal model in subsequent experiments.

### 4.2 Decreased CKB Resulted in Hippocampal Structural Plasticity Deficiency and Learning and Memory Impairments

Many research efforts have underscored the pivotal function of CK‐BB in conditions affecting the nervous system, such as neurodegenerative and psychiatric disorders (Aksenov et al. 1997; Burbaeva, Savushkina, and Dmitriev 1999; Jost et al. 2002). A reduction in CK‐BB activity might disrupt the balance of cellular energy, leading to damage within the central nervous system (Laakso et al. 2003). Burbaeva and colleagues documented that the concentration of cytosolic CK‐BB in brain samples from individuals who had AD or schizophrenia, and who had undergone autopsy, was considerably lower compared to levels found in analogous control brain samples (Burbaeva, Savushkina, and Dmitriev 1999). In individuals with AD, a drop in overall CK activity was linked to a decrease in CK‐BB activity, even though the expression levels of CK‐BB remained largely unchanged (Aksenov et al. 1997). Mice lacking CK‐BB exhibited signs of compromised energy preservation, weakened connections in the hippocampal mossy fiber pathways, reduced adaptability in open‐field environments, and a deterioration in memory functions associated with aging (Aksenov et al. 1997). However, research examining the expression levels of CK‐BB in the elderly and in animal models is scarce. Additionally, the precise pathophysiological impact of CK‐BB on the process of brain aging remains to be established. In this study, we utilized microarray data from the GEO database to identify DEGs in brain tissues from individuals with cognitive impairments, AD, or dementia. Our analysis revealed that the CKB gene was downregulated in samples from AD patients (Figure 1A). Our study further showed that CK‐BB activity and protein level decreased significantly after injections of 100 and 1000 mg/kg D‐gal (Figure 1G–I). However, direct evidence explaining whether the decrease in CK‐BB content was responsible for the structural plasticity and behavioral consequences of long‐term D‐gal administration does not exist.

Therefore, we produced a mouse model without CK‐BB expression in the hippocampus through CKB mRNA knockdown to specifically investigate the effects of CK‐BB on hippocampal structural plasticity and cognitive behavior. Our results showed that similar to those in mice that received repeated D‐gal injections, the protein level and activity of CK‐BB decreased significantly in the AAV‐shCKB group (Figure 3C–E). Subsequently, we analyzed the functional consequences of the absence of hippocampal CK‐BB. We found that the knockdown of CK‐BB in the hippocampus induced cognitive deficits, oxidative stress, and hippocampal structural plasticity impairment. These data suggest that the loss of CK‐BB function would likely produce the same effects on hippocampal morphological plasticity as the long‐term injection of D‐gal. We speculated that downregulated of CK‐BB in hippocampus led to the imbalance of cellular energy which disturbed the metabolic of nerve cells, further damage the formation of synapses, and eventually cause behavioral disorders.

### 4.3 Cr Supplementation Ameliorated Cognitive Deficits and Hippocampal Structural Plasticity Impairment by Increasing CK‐BB Activity and Expression

Brain Cr is inversely correlated with age and can be enhanced through memory exercises (Solis et al. 2017; Valenzuela et al. 2003). Studies have shown that Cr has a protective effect on the brain, as evidenced by its positive impact in animal models of neurodegenerative conditions such as Huntington's and Parkinson's diseases (Andres, Wallimann, and Widmer 2016; Leem et al. 2024). These observations suggested a significant link between the CK/pCr/Cr system's efficiency and the brain's proper functioning. In our research, we discovered that a 3% dietary Cr supplement eliminated the decline in CK‐BB activity and protein levels caused by prolonged D‐gal exposure. Notably, in mice, Cr supplementation significantly relieved learning and memory deficits and reduced oxidative stress compared with chronic D‐gal injection (Figure 4, Figure S3). The process of aging correlates with reduced amounts of creatine and phosphocreatine, particularly in skeletal muscles. The intake of creatine supplements can lead to an increase in both creatine and phosphocreatine levels by 10%–40% in athletes (Kreider 2003). Given that the creatine transporter efficiently moves creatine from the blood into the brain across the blood–brain barrier (BBB), it is logical to propose that the addition of creatine from external sources would raise its levels in the brain, where natural levels of creatine may decrease with age. In the context of our results, creatine supplementation could alleviate D‐gal‐induced cognitive impairments, creatine may be used as a health supplement for the elderly which contributed to a slowdown in the advancement of these conditions. The results present a new approach to postponing neurodegenerative diseases and enhancing memory and cognitive abilities.

Our results show that supplementation with 3% Cr also reversed hippocampal structural plasticity impairment after repeated D‐gal administration. Creatine is reported to boost energy supply, muscle power, total protein synthesis, and the differentiation of muscle‐forming cells in muscles affected by dystrophy (Deldicque et al. 2007; McAleer et al. 2014). Synapse formation is an energy‐consuming process that requires protein synthesis. Our above data suggest that Cr supplementation improved cognition by activating and increasing the activities and expression of CK‐BB. It is possible that creatine supplementary could increase the expression and activity of CK‐BB which contribute to the new synapse formation.

Considering the metabolic disruptions that occur in the aging brain, Cr supplementation could be advantageous in rectifying disrupted energy metabolism by replenishing energy sources, stimulating mitochondrial respiration, and safeguarding cells from apoptosis (Dolder et al. 2003; Kay et al. 2000; O'Gorman et al. 1997). In young, physically active adults, Cr intake during resistance training may enhance muscle strength and endurance (Mills et al. 2020). Thus, the actual role of Cr in energy metabolism warrants further investigation. CK‐BB is recognized as a key target for oxidative damage, a characteristic feature of many neurodegenerative diseases (Aksenov et al. 2000). Consequently, increasing intracellular Cr levels through supplementation may provide extra protection to CK, thereby delaying the inactivation of the CK system in the brain due to free radicals. It is well known that under hypoxic conditions, the pH of cells decreases and acidic products increase, promoting oxidative stress responses. A recent study investigating a single dose of creatine on sleep deprivation, creatine was shown to prevent pH drop and acidosis during sleep deprivation (Gordji‐Nejad et al. 2024). This is consistent with our results that creatine supplementation could alleviated oxidant stress index induced by D‐gal in mice.

The study's constraint lies in the unclear precise mechanism through which CK‐BB specifically manages cognitive impairment caused by aging. Our findings indicate that increased CK‐BB levels, achieved through creatine supplementation, can ameliorate cognitive deficits triggered by D‐gal. CK‐BB is localized in the mitochondria, which are also the primary site for energy metabolism. Possible ways this might occur include shielding against oxidative stress or maintaining mitochondrial membrane potential; modulating Na+/K+‐ATPase and/or CAMKII/CREB pathways. CK‐BB might modulate mitochondrial energy metabolism, thereby affecting oxidative reactions to enhance synaptic plasticity and ultimately manage cognitive impairment. However, these mechanisms are not well comprehended and further investigation is needed to explore the diverse effects of creatine on brain function and cognitive processing. Additionally, a limitation in this study is the reduction of CK‐BB expression by injecting AAV‐shCKB into the hippocampus. The involvement of CK‐BB in cognitive dysfunction could be more clearly defined using conditional knockout models.

In conclusion, our findings support previous research highlighting the critical role of CK‐BB in modulating neuronal structural plasticity and cognitive functions. A 3% Cr supplement improved learning, memory, and neuronal structural plasticity in aging mice, suggesting that Cr supplementation could be a promising nutritional strategy for enhancing cognitive health in aging individuals.

## Author Contributions

**Zhu Zhu:** writing – original draft (lead). **Hantao Zhang:** methodology (lead). **Qianlin Li:** methodology (equal). **Xu Han:** data curation (equal). **Tiantian Wang:** validation (equal). **Wenjing Zhang:** methodology (supporting). **Feiyan Chen:** funding acquisition (equal). **Ling Gu:** software (equal). **Qi Yao:** supervision (equal). **Lin Chen:** funding acquisition (equal), supervision (equal). **Yunan Zhao:** project administration (equal), supervision (equal), writing – review and editing (equal).

## Conflicts of Interest

The authors declare no conflicts of interest.

## Supporting information


Appendix S1

Figure S1

Figure S2

Figure S3

## Data Availability

The data that support the findings of this study are available on request from the corresponding author. The data are not publicly available due to privacy or ethical restrictions.
