# Supplementary material for: Long‐Term Creatine Supplementation Improves Cognitive and Hippocampal Structural Plasticity Impairments in a D‐Gal‐Induced Aging Model via Increasing CK‐BB Activity in the Brain
Source: Food Sci Nutr. 2025 Jan 15;13(1):e4767. doi: 10.1002/fsn3.4767 (PMC11733680; doi:10.1002/fsn3.4767)
Supplement: Supplementary file 1 — Appendix S1 [file FSN3-13-e4767-s002.docx]

**Supplementary Information**

**Supplementary Results**

Experiment 2: Effects of CK-BB knockdown in the hippocampus on learning and memory, oxidative stress, and the structural plasticity of hippocampal neurons

*Silencing efficiency of the shRNAs against CKB in the hippocampi of mice.* Two-way ANOVA showed significant changes in CK-BB expression, with the effects of shCKB (F_1, 4_ = 39.44, p = 0.0033) and D-gal and shCKB interaction (F_1, 4_ = 14.78, p = 0.0184) being significant. However, D-gal (F_1, 4_ = 5.336, p = 0.082) did not have a significant effect on CK-BB expression. In addition, D-gal (F_1, 5_ = 8.164, p = 0.0355) had a significant effect on CK-BB activity in contrast to shCKB (F_1, 5_ = 3.186, p = 0.1343) and the interaction between the two factors (F_1, 5_ = 3.264, p = 0.1306).

*Knockdown of CK-BB in the hippocampus induced cognitive dysfunction and oxidative stress in mice.* Two-way ANOVA revealed the significant effects of D-gal (F_1, 5_ = 7.771, p = 0.0386) and the interaction between D-gal and shCKB (F_1, 5_ = 11.17) on the time spent in the target quadrant, with shCKB lacking a significant effect (F_1, 5_ = 6.381, p = 0.0528). The number of platform crossings was affected by D-gal (F_1, 6_ = 8.000, p = 0.03), shCKB (F_1, 6_ = 10.5, p = 0.0177) and the interaction between these two factors (F_1, 6_ = 29.54, p = 0.0016). In the Y-maze test, the effects of D-gal (F_1, 6_ = 7.111, p = 0.0372) and shCKB (F_1, 6_ = 6.439, p = 0.0422) on the ratio of entries into the novel arm to entries into the familiar arm were significant, whereas that of the interaction between the two factors was not (F_1, 6_ = 2.345, p = 0.1766). For SOD activity, an oxidative stress biomarker, the effects of D-gal (F_1, 3_ = 15.95, p = 0.0281) and shCKB (F_1, 3_ = 19.81, p = 0.0211) were significant, whereas that of the interaction between the two factors (F_1, 3_ = 1.676, p = 0.286) was not. Two-way ANOVA showed that the effect of shCKB (F_1, 5_ = 6.861, p = 0.0471) on GSH-Px activity was significant, whereas those of D-gal (F_1, 5_ = 4.836, p = 0.0792) and the interaction between these two factors (F_1, 5_ = 5.275, p = 0.0701) were not. The effects of D-gal (F_1, 4_ = 15.16, p = 0.0176) and the interaction between D-gal and shCKB (F_1, 4_ = 10.86, p = 0.0301) on MDA levels were significant, whereas that of shCKB (F_1, 4_ = 5.700, p = 0.0754) was not.

*Knockdown of CK-BB impaired the hippocampal structural plasticity of neurons in mice.* Two-way ANOVA revealed that D-gal (PSD-95: F_1, 4_ = 101.3, p = 0.0005; NF-L: F_1, 4_ = 25.93, p = 0.007; BNDF: F_1, 4_ = 136.7, p = 0.0003), shCKB (PSD-95: F_1, 4_ = 28.63, p = 0.059; NF-L: F_1, 4_ = 11.74, p = 0.0266; BDNF: F_1, 4_ = 302.5, p < 0.0001), and the interaction between these two factors (PSD-95: F_1, 4_ = 9.073, p = 0.0395; NF-L: F_1, 4_ = 11.20, p = 0.0287; BDNF: F_1, 4_ = 117.3, p = 0.0004) had significant effects on the protein levels of PSD-95, NF-L, and BDNF. Two-way ANOVA illustrated that D-gal (F_1, 7_ = 10.26, p = 0.015) had a significant effect on hippocampal spine density in CA1, whereas the effects of shCKB (F_1, 7_ = 2.277, p = 0.175) and the interaction between the two factors (F_1, 2_ = 7.194, p = 0.1154) were not significant. The effects of D-gal (F_1, 42_ = 36.52, p < 0.0001), shCKB (F_1, 42_ = 53.16, p < 0.0001), and the interaction between the two factors (F_1, 42_ = 16.32, p = 0.0002) on the total dendritic length of hippocampal neurons in CA1 were significant.

Experiment 3. Cr supplementation improved cognitive deficits, oxidative stress, and the structural plasticity of hippocampal neurons in the D-gal-induced aging mouse model by increasing the activity and protein levels of CK-BB

*Supplementary Cr in the diet relieved learning and memory deficits and oxidative stress in mice.* Two-way ANOVA revealed that D-gal (F_1, 7_ = 8.125, p = 0.0247) and Cr (F_1, 7_ = 10.01, p = 0.0158) had significant effects on the time spent in the target quadrant, whereas the interaction between the two factors (F_1, 7_ = 4.164, p = 0.0806) had no significant effects. The effect of D-gal (F_1, 7_ = 11.20, p = 0.0123) on the the number of platform crossings was significant, whereas those of Cr (F_1, 7_ = 3.706, p = 0.0956) and the interaction between the two factors (F F_1, 7_ = 0.3043, p = 0.5983) were not. In the Y-maze test, two-way ANOVA showed that D-gal (F_1, 7_ = 6.677, p = 0.0363) and Cr (F_1, 7_ = 5.971, p = 0.0455) had a significant effect on the ratio of entries into the novel arm to entries into the familiar arm, whereas the interaction between the two factors (F_1, 7_ = 4.278, p = 0.0774) did not. The effects of Cr (F_1, 4_ = 23.80, p = 0.0082) and the interaction between D-gal and Cr (F_1, 4_ = 9.720, p = 0.0356) on SOD activity, an oxidative stress biomarker, were significant, whereas that of D-gal (F_1, 4_ = 4.468, p = 0.102) was not. Two-way ANOVA showed that the interaction between D-gal and Cr (F_1, 4_ = 9.858, p = 0.0349) had a significant effect on GSH-Px activity, whereas D-gal (F_1, 4_ = 2.634, p = 0.1799) and Cr (F_1, 4_ = 3.765, p = 0.1243) did not. Cr (F_1, 4_ = 12.72, p = 0.0234) had a significant effect on MDA level, whereas D-gal (F_1, 4_ = 6.059, p = 0.0696) and the interaction between the two factors (F_1, 4_ = 3.197, p = 0.1483) did not.

*Cr supplementation increased the activity and protein level of CK-BB in the hippocampi of mice.* Two-way ANOVA showed that D-gal (F_1, 4_ = 93.04, p = 0.0006) and Cr (F_1, 4_ = 11.31, p = 0.0282) had significant effects on CK-BB activity, whereas the interaction between the two factors (F_1, 4_ = 0.3245, p = 0.5994) did not. In addition, the interaction between D-gal and Cr (F_1, 4_ = 11.55, p = 0.0273) had a significant effect on CK-BB expression, whereas D-gal (F_1, 4_ = 3.626, p = 0.1296) and Cr (F_1, 4_ = 0.1823, p = 0.6914) did not.

*Cr supplementation improved hippocampal structural plasticity.* Two-way ANOVA showed that Cr (F_1, 4_ = 41.21, p = 0.003) had a significant effect on the protein level of PSD-95, whereas D-gal (F_1, 4_ = 3.613, p = 0.1301) and the interaction between the two factors (F_1, 4_ = 0.4754, p = 0.5284) did not. Furthermore, the effects of D-gal (F_1, 4_ = 14.04, p = 0.02) and Cr (F_1, 4_ = 27.37, p = 0.0064) were significant, whereas that of the interaction between the two factors (F_1, 4_ = 2.939, p = 0.1616) was not. The effect of D-gal (F_1, 4_ = 12.02, p = 0.0257) on the protein level of BDNF was significant, whereas those of Cr (F_1, 4_ = 4.283, p = 0.1073) and the interaction between the two factors (F_1, 4_ = 0.8150, p = 0.4177) were not. Two-way ANOVA demonstrated that Cr (F_1, 15_ =32.7, p < 0.0001) and the interaction between D-gal and Cr (F_1, 15_ = 21.35, p = 0.0003) had significant effects on hippocampal spine density in CA1, whereas D-gal (F_1, 15_ = 1.975, p = 0.1803) did not. The effects of D-gal (F_1, 9_ = 18.31, p = 0.0021), Cr (F_1, 9_ = 33.57, p = 0.0003), and the interaction between the two factors (F_1, 9_ = 11.90, p = 0.0073) had significant effects on the total dendritic length of hippocampal neurons in CA1.

Figure legend

Fig.S1. The effects of different doses of D-gal on oxidative stress. (A-C) Antioxidant indexes in serum. (A) Activity levels of SOD and (B) GSH-Px. (C) Concentration of MDA. *p < 0.05, **p < 0.01 and p < 0.001 vs control group; mean ± SEM.

Fig.S2. Effects of CK-BB knockdown in the hippocampus on oxidant stress. (A−C) Antioxidant indexes in serum. (A) Activity levels of SOD and (B) GSH-Px. (C) Concentration of MDA. *p < 0.05, **p < 0.01 and p < 0.001 vs control group; mean ± SEM.

Fig.S3. Effects of Cr supplementation on oxidant stress. (A−C) Antioxidant indexes in serum. (A) Activity levels of SOD and (B) GSH-Px. (C) Concentration of MDA. *p < 0.05, **p < 0.01 and p < 0.001 vs control group; #p < 0.05, ##p < 0.01 vs D-gal group; mean ± SEM.
