# Supplementary figures and images for: Long‐Term Creatine Supplementation Improves Cognitive and Hippocampal Structural Plasticity Impairments in a D‐Gal‐Induced Aging Model via Increasing CK‐BB Activity in the Brain
Source: Food Sci Nutr. 2025 Jan 15;13(1):e4767. doi: 10.1002/fsn3.4767 (PMC11733680; doi:10.1002/fsn3.4767)

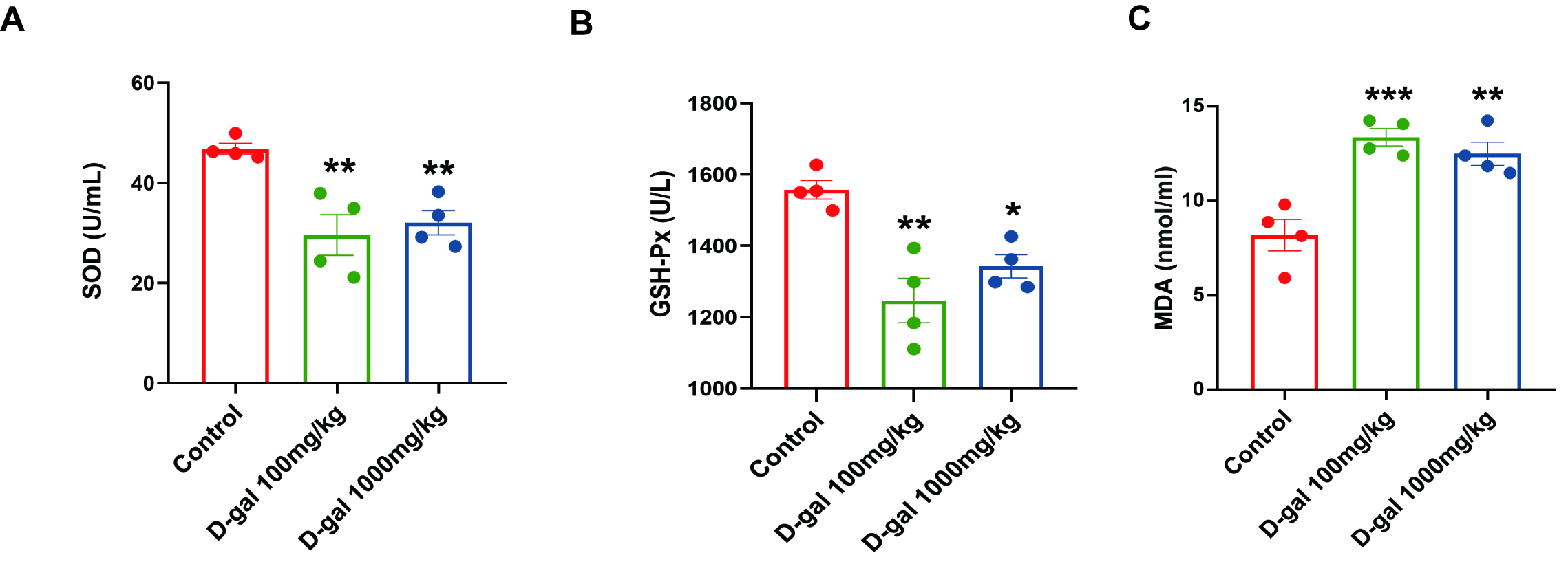

Supplement: Supplementary file 2 — Figure S1 [file FSN3-13-e4767-s001.tif]

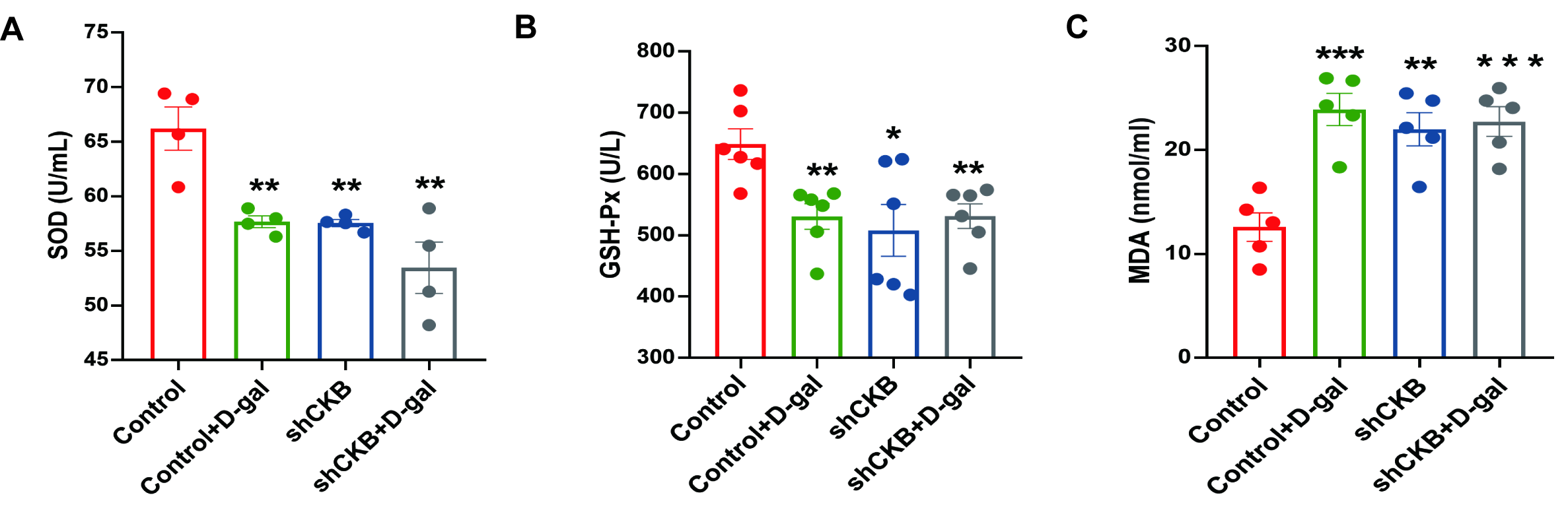

Supplement: Supplementary file 3 — Figure S2 [file FSN3-13-e4767-s004.tif]

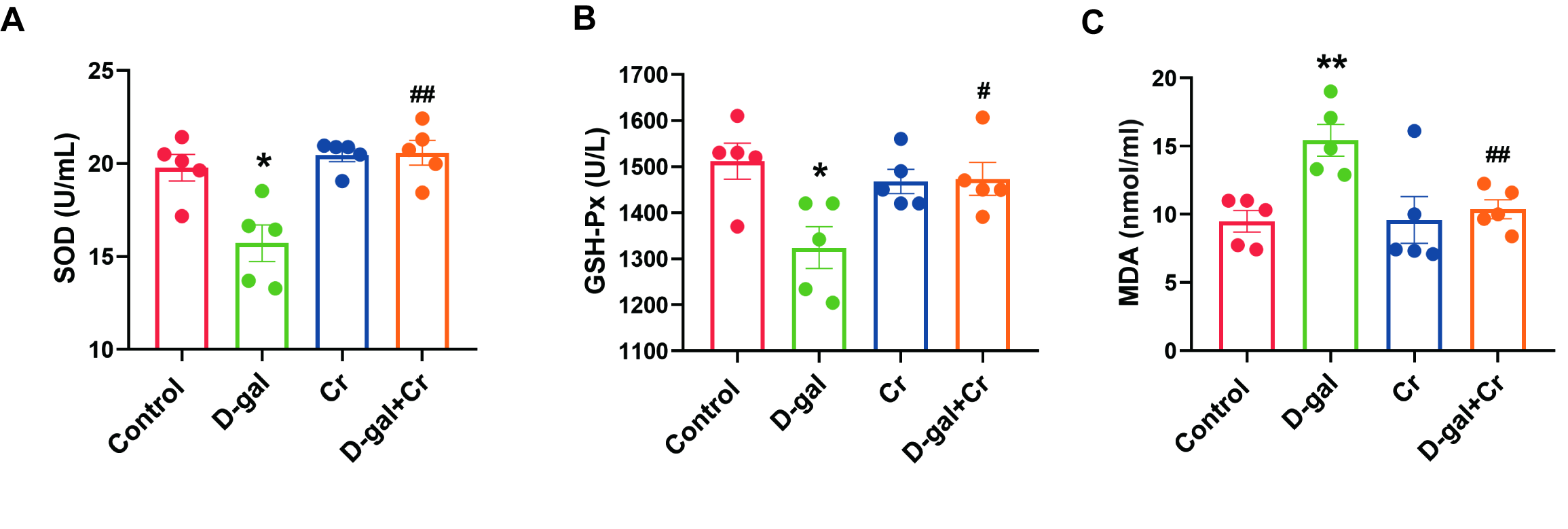

Supplement: Supplementary file 4 — Figure S3 [file FSN3-13-e4767-s003.tif]
